# Pharmacovigilance in juvenile idiopathic arthritis patients (Pharmachild) treated with biologic agents and/or methotrexate. Consolidated baseline characteristics from Pharmachild and other national registries

**DOI:** 10.1186/1546-0096-12-S1-P7

**Published:** 2014-09-17

**Authors:** Joost Swart, Alessandro Consolaro, Gerd Horneff, Kimme L Hyrich, Francesca Bovis, Jose Melo-Gomes, Ekaterina Alexeeva, Stefano Lanni, Gerd Ganser, Violeta Panaviene, Jordi Anton, Ivan Foeldvari, Valda Stanevicha, Susan Nielsen, Ralf Trauzeddel, Constantin Ailioaie, Pierre Quartier, Toni Hospach, Gordana Susic, Maria Trachana, Frank Weller-Heinemann, Alberto Martini, Nico Wulffraat, Nicolino Ruperto

**Affiliations:** 1 UMC Utrecht, Utrecht, Netherlands; 2 Istituto Giannina Gaslini, Genoa, Italy; 3 Zentrum für Allgemeine Paediatrie und Neonatologie - Asklepios Klinik Sankt Augustin, Sankt Augustin, Germany; 4 Arthritis Research UK Centre for Epidemiology, University of Manchester, Manchester, UK; 5 St. Josef-Stif Sendenhorst, Sendenhorst, Germany; 6 Hamburger Zentrum für Kinder- und Jugendrheumatologie, Hamburg, Germany; 7 Helios Kliniken Berlin, Berlin; 8 Olga Hospital, Stuttgart, Germany; 9 Klinikum Bremen-Mitte, Bremen, Germany

## Introduction

The availability of methotrexate (MTX) and biological agents has provided a major change in the treatment of children with juvenile idiopathic arthritis (JIA). An international registry named Pharmachild (European Union grant 260353) has been set up by the Pediatric Rheumatology International Trials Organisation (PRINTO)/Pediatric Rheumatology European Society (PRES). In parallel several national registries with the same purpose have been set up in different European Countries for the follow-up of these patients.

## Objectives

To evaluate the possibility to combine data coming from different national registries in order to assess the long-term safety.

## Methods

We merged into a unified database the baseline demographic data of JIA patients treated with MTX or biologicals coming from the Pharmachild registry and from the national registries of Germany, United Kindgom and Portugal. Data are presented as frequencies (%) or medians with 1st and 3rd quartiles.

## Results

About 63% of the patients has been treated with biologicals alone or in combination with MTX, and 29% only with MTX. The events of special interest (ESI) ranged from 4.5 to 15.0% and the other moderate/severe/serious adverse events (AE) from 13.1% to 69.9%. Table 1.

**Table 1:** 

	Pharmachild (N=5571)	NR UK (N=1537)	NR Germany (N=3243)	NR Portugal (N=112)	TOTAL (N=10463)
**Age at onset**	5.4 (2.4 – 10.0)	-	7.2 (3.1-11.4)	6.3 (2.5-10.9)	-

**Age at JIA Diagnosis**	6.2 (2.8 – 11.0)	5.5 (2.1-10.2)	8.2 (4.0-12.3)	7.3 (3.3-12.3)	-

**Disease duration at the last follow-up**	4.9 (2.5 – 8.2)	5.4 (2.7 – 8.8)	5.2 (3.1-8.4)	3.0 (0.5-9.6)	-

**Therapy with MTX only**	1365 (24.5)	503 (32.7)	1132/3134 (36.1)	0 (0.0)	3000/10354 (29.0)

**Therapy with only one Biologic Drug + MTX**	2378 (42.7)	862 (56.1)	1545/3134 (49.2)	27 (24.1)	4812/10354 (46.5)

**Therapy with more than one Biologic + MTX**	872 (15.6)	141 (9.2)	340/3134 (10.8)	78 (69.6)	1431/10354 (13.8)

**Nr. patients with ESI or AE**	1070 (19.2)	1093 (71.1)	1163(37.1)	27 (24.1)	3353 (32.4)

**Nr. patients with ESI**	496 (8.9)	230 (15.0)	249 (7.9)	5 (4.5)	980 (9.5)

**Nr. patients with AE**	729 (13.1)	1075 (69.9)	1069 (34.1)	24 (21.4)	2897 (28.0)

## Conclusion

Combination of information from different data sources is a recommended task and will provide a powerful tool for the future analysis of safety events coming from different registries.

## Disclosure of interest

None declared.

